# Energy-Efficient Smart Window Based on a Thermochromic Hydrogel with Adjustable Critical Response Temperature and High Solar Modulation Ability

**DOI:** 10.3390/gels10080494

**Published:** 2024-07-25

**Authors:** Meng Sun, Hui Sun, Ruoyu Wei, Wenqing Li, Jinlai Lai, Ye Tian, Miao Li

**Affiliations:** 1 Key Laboratory of Environment Controlled Aquaculture, (Dalian Ocean University) Ministry of Education, Dalian 116023, China; sunmeng04262022@163.com (M.S.); sh180089@163.com (H.S.); 2 College of Marine Technology and Environment, Dalian Ocean University, Dalian 116023, China; weiruoyu0516@163.com (R.W.); liwenqing052626@163.com (W.L.); laijinlai5410@163.com (J.L.); 3 College of Biosystems Engineering and Food Science, Zhejiang University, Hangzhou 310058, China; 4 School of Biological Engineering, Dalian Polytechnic University, Dalian 116034, China; limiao@dlpu.edu.cn

**Keywords:** thermochromic hydrogel, smart window, solar modulation, adjustable response temperature

## Abstract

Thermochromic smart windows realize an intelligent response to changes in environmental temperature through reversible physical phase transitions. They complete a real-time adjustment of solar transmittance, create a livable indoor temperature for humans, and reduce the energy consumption of buildings. Nevertheless, conventional materials that are used to prepare thermochromic smart windows face challenges, including fixed transition temperatures, limited solar modulation capabilities, and inadequate mechanical properties. In this study, a novel thermochromic hydrogel was synthesized from 2-hydroxy-3-butoxypropyl hydroxyethyl celluloses (HBPEC) and poly(N-isopropylacrylamide) (PNIPAM) by using a simple one-step low-temperature polymerization method. The HBPEC/PNIPAM hydrogel demonstrates a wide response temperature (24.1–33.2 °C), high light transmittance (*T*_lum_ = 87.5%), excellent solar modulation (Δ*T*_sol_ = 71.2%), and robust mechanical properties. HBPEC is a functional material that can be used to adjust the lower critical solution temperature (LCST) of the smart window over a wide range by changing the degree of substitution (DS) of the butoxy group in its structure. In addition, the use of HBPEC effectively improves the light transmittance and mechanical properties of the hydrogels. After 100 heating and cooling cycles, the hydrogel still has excellent stability. Furthermore, indoor simulation experiments show that HBPEC/PNIPAM hydrogel smart windows have better indoor temperature regulation capabilities than traditional windows, making these smart windows potential candidates for energy-saving building materials.

## 1. Introduction

In recent years, the severity of both global warming and the energy crisis has escalated significantly. Consequently, there is a growing urgency for energy-saving and emission-reduction strategies [1,2]. Carbon dioxide emissions from buildings contribute to nearly 40% of direct and indirect global emissions, and thus, numerous strategies have been proposed to address this critical issue [3,4]. Specifically, windows are an important part of buildings, but they are also a significant component of indoor–outdoor heat exchange. Frequent heat exchange leads to higher indoor energy consumption. Therefore, minimizing energy consumption that is due to windows is essential for enhancing building efficiency and achieving energy savings. Hence, smart windows offer a practical and promising approach to emission reduction in sustainable building design [5,6].

At present, there are four main types of prevailing smart window technologies: thermochromic [7], photochromic [8], mechanical [9], and electrochromic [10,11,12,13]. Of these, thermochromic smart windows passively modulate light transmittance according to the temperature of the surroundings without extra energy consumption. This feature has significant potential for energy-saving applications in the building sector [14,15]. Among the many thermochromic materials, VO_2_, perovskites, and hydrogels have been the most intensively studied [16,17,18]. Hydrogel materials exhibit lower phase transition temperatures and exhibit a broad spectrum of adjustable chemical and physical properties compared to VO_2_, perovskites, and other inorganic thermochromic materials [19]. Consequently, hydrogels have great application potential in the field of smart windows [20,21,22].

PNIPAM is a common hydrogel thermochromic material that undergoes a physical phase transition at lower temperatures (~32 °C) and has impressive solar modulation capabilities [23]; thus, it a prime choice for smart window materials [24]. Nevertheless, the PNIPAM-based thermochromic hydrogel has limitations, including low light transmittance, weak mechanical strength, and a fixed phase transition temperature that is not adjustable for diverse regional requirements. Hence, many researchers have explored a variety of strategies for addressing these challenges. For example, Wei et al. [25] enhanced the solar radiation modulation capacity of the hydrogel by introducing Ag nanorods (NRs). The resulting PNIPAM-acrylic/Ag NRs hybrid hydrogel exhibited a Δ*T*_sol_ of 59.24% and *T*_lum_ of 61.36% at room temperature and maintained good cyclic stability. Although PNIPAM-acrylic/Ag NRs hydrogels exhibit enhanced properties, there is still potential for further improvement in terms of Δ*T*_sol_ and *T*_lum_. Introducing hydrophilic monomers can increase the hydrogen bonding interactions within the molecular chains as well as between molecular chains and water molecules to enhance the mechanical properties and transmittance of hydrogels. Chen et al. [26] introduced hydrophilic 4-acryloyl morpholine (ACMO) vinyl monomers into NIPAM, and the resulting smart window exhibited an impressive *T*_lum_ of 85.847% and ∆*T*_sol_ of 79.332%. However, the phase transition temperature remained fixed.

In recent years, researchers have introduced amphiphilic alkyl cellulose ethers into thermochromic materials. These ethers enhance the transmittance of the hydrogel and give the material temperature-responsive properties [27,28,29]. However, it is important to recognize that the LCST of these cellulose derivatives is high and challenging to control. The literature suggests that the LCST of cellulose-based hydrogels can only be modified by introducing supplementary substances [30]. In our previous work [31], we introduced alkyl side chains that had varying carbon chain lengths into the main hydroxyethyl cellulose backbone. By adjusting the carbon chain length and degree of substitution, we achieved a hydrophilic–hydrophobic balance in the alkylated modified hydroxyethyl cellulose (HEC). Consequently, we obtained a series of cellulose ether temperature-sensitive materials that exhibited rapid responses to temperature changes, and their LCST could be precisely regulated within a specific temperature range. Thus, these materials are potential candidates for use in thermochromic smart windows.

In this work, a thermochromic hydrogel material (HBPEC/PNIPAM) that had high light transmittance (*T*_lum_ = 87.5%), excellent solar modulation (Δ*T*_sol_ = 71.2%), and good mechanical properties was prepared using the “one-pot integration tactics” integration strategy with HBPEC and NIPAM as raw materials. The application of the hydrogel in smart windows was then studied. The thermochromism of the hydrogel results from HBPEC and PNIPAM, but the regulation of LCST only depends on HBPEC. When the degree of butyl substitution in HBPEC is in the range of 0.98–2.32, the LCST of the HBPEC/PNIPAM hydrogel can be regulated in the range of 24.1–33.2 °C. When the temperature is lower than the LCST, the hydrogel is transparent, which would allow sunlight to enter a room in which such windows were used. When the temperature is higher than the LCST, the hydrogel undergoes a phase transition from transparent to opaque, and this would block sunlight from entering the room. Additionally, in simulated indoor temperature regulation tests, the smart window exhibits satisfactory solar cooling capabilities. Compared to ordinary windows, the HBPEC/PNIPAM smart window offers excellent and stable indoor temperature regulation, leading to an effective reduction in building energy consumption and resulting in significant energy savings for buildings.

## 2. Results and Discussion

### 2.1. Fabrication of the HBPEC/PNIPAM Hydrogel

**Scheme 1:**
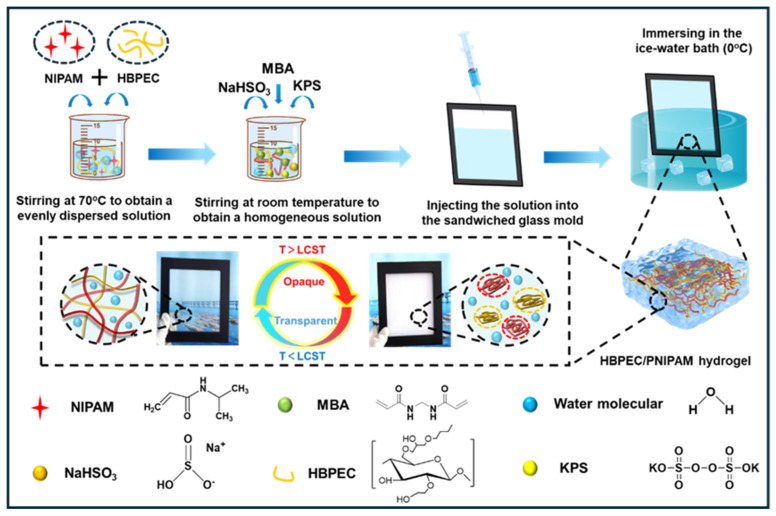
The phase transformation mechanism diagram and preparation process of HBPEC/PNIPAM hydrogels.

Scheme 1 illustrates the phase transformation mechanism diagram and preparation process of HBPEC/PNIPAM hydrogels. A simple “one-pot” integration strategy involving low-temperature radical polymerization was employed to synthesize HBPEC/PNIPAM hydrogels. This approach offers benefits such as simple preparation, rapid polymerization, precise structural control, and adaptability to different monomers. The incorporation of HBPEC and NIPAM as raw materials improves both the transparency and mechanical properties of the hydrogel. Notably, the addition of HBPEC can regulate the LCST of the hydrogel, which solves the limitation that the traditional PNIPAM hydrogel can only undergo a physical phase transition at 32 °C.

First, the precursor solution was prepared by mixing the monomers NIPAM and HBPEC. Then, in a redox system composed of KPS and NaHSO_3_, a three-dimensional hydrophilic hydrogel network was formed via low-temperature free radical in situ polymerization; this resulted in a series of HBPEC/PNIPAM hydrogels with high light transmittance, excellent solar modulation ability, and outstanding mechanical properties. A large amount of heat is released during the polymerization process, and thus, the polymer causes molecular chain shrinkage at high temperatures, which seriously affects the transparency of the hydrogel [32]. Therefore, the synthesis reaction is carried out in an ice water bath, which can disperse the heat in the polymerization process in time and effectively improve the transmittance of the hydrogel. To study the effects that HBPEC components with different DS have on the properties of HBPEC/PNIPAM hydrogels, the prepared hydrogels were labeled as H_x_/P hydrogels, where x is the DS of HBPEC. The hydrogels that were prepared with the addition of HBPEC with different DS are shown in Table 1.

**Scheme 2:**
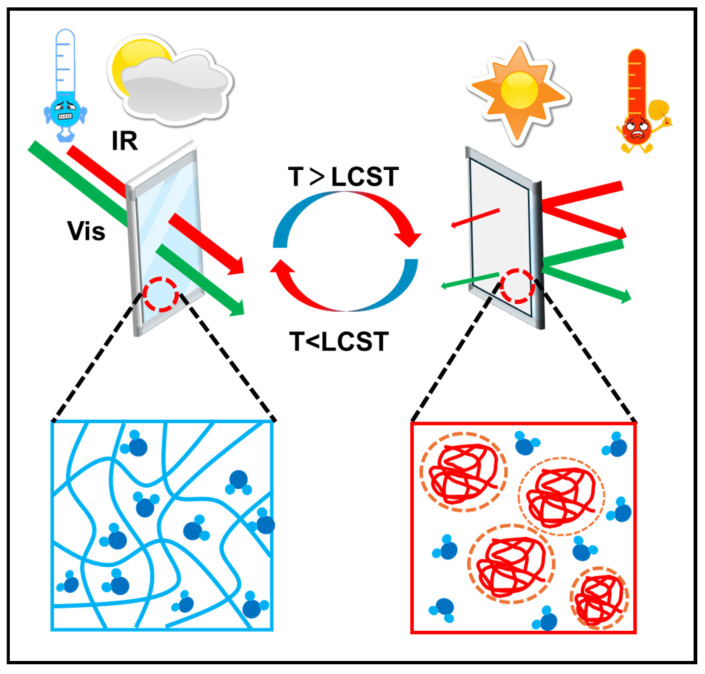
Phase transition mechanism diagram of the HBPEC/PNIPAM hydrogel.

Scheme 2 shows the thermochromic mechanism of the HBPEC/PNIPAM hydrogel as it transitions from a transparent state to an opaque state around the LCST. Adjusting the hydrophilic–hydrophobic balance along the polymer molecular chain allows the hydrogel to exhibit various states. When the environmental temperature falls below the LCST, strong hydrogen bonds form between the hydrophilic groups on the polymer chain and the surrounding water molecules, and this renders the hydrogel transparent. The intermolecular hydrogen bonds in the internal structure enable sunlight to penetrate the material. Conversely, when the environmental temperature is above the LCST, the interaction between hydrophobic groups is enhanced. When this happens, the intermolecular hydrogen bonds in the internal structure of the polymer break and form intramolecular hydrogen bonds, resulting in the aggregation of the polymer to form a hydrophobic region. This condition makes the hydrogel opaque and prevents the penetration of solar light, thereby achieving solar cooling.

**Figure 1:**
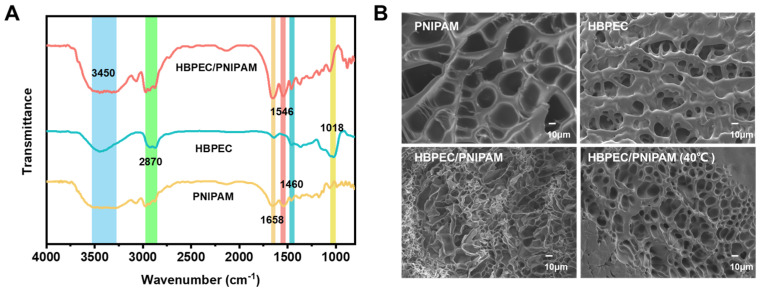
(**A**) FTIR spectra of PNIPAM, HBPEC, and HBPEC/PNIPAM hydrogels. (**B**) SEM images of PNIPAM, HBPEC, and HBPEC/PNIPAM hydrogels.

The chemical compositions of PNIPAM, HBPEC, and HBPEC/PNIPAM hydrogels were verified using Fourier transform infrared (FTIR) spectroscopy. As seen in Figure 1A, the broad peak at 3450 cm^−1^ is related to the O-H stretching of the structural water and the N−H stretching vibration of PNIPAM. The three characteristic absorption peaks at 1658 cm^−1^, 1546 cm^−1^, and 1460 cm^−1^ are attributed to amide I (C = O tensile vibration), amide II (N-H bending vibration), and amide III (C-N tensile vibration), respectively. The absorption peaks of HBPEC at 2917 cm^−1^ and 1018 cm^−1^ are attributed to the stretching vibration of C-H bonds and vibration of the N-H plane deformation. The FTIR spectrum of the HBPEC/PNIPAM hydrogel shows simultaneous characteristic absorption peaks for HBPEC and PNIPAM, and this confirms that the HBPEC/PNIPAM composite hydrogel was successfully synthesized.

SEM was used to observe the morphologies of PNIPAM, HBPEC, and HBPEC/PNIPAM. As seen in Figure 1B, the surface of the pure PNIPAM hydrogel exhibited a network structure that was crossed with irregular holes. Upon adding HBPEC, a more densely woven fibrous network structure formed within the HBPEC/PNIPAM hydrogel. This phenomenon can be attributed to the abundant hydrophilic groups in HBPEC, facilitating hydrogen bonding with water and cross-linking to enhance structural density. When the temperature was 40 °C, the porous fibrous structure of the HBPEC/PNIPAM hydrogel began to aggregate and shrink rapidly; this is why the hydrogel changed from a transparent state to an opaque state.

### 2.2. Tuning the LCST of HBPEC/PNIPAM Hydrogels by Adjusting the Butoxy DS of HBPEC

**Figure 2:**
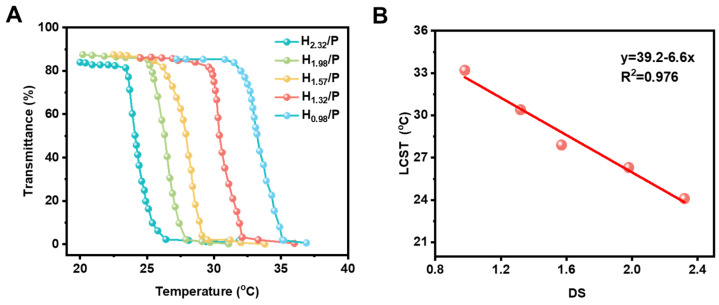
(**A**) Transmittance of H_0.98–2.32_/P hydrogels as a function of temperature. (**B**) Effects that HBPEC/PNIPAM hydrogels with different DS have on LCST.

Freely tunable LCST are key factors affecting the application of thermochromic smart windows [33,34]. The LCST of a hydrogel is related to the hydrophilicity of the hydrogel. Specifically, the LCST increases with an increase in the hydrophilicity of a hydrogel, whereas it decreases with an increase in the hydrophobicity of hydrogels [35]. We control the LCST of the HBPEC/PNIPAM hydrogel by varying the butoxy DS of the HBPEC component. The thermochromic properties of the hydrogel originate from HBPEC and PNIPAM, but LCST regulation is solely determined by the HBPEC component. With higher butoxy DS, HBPEC exhibits greater hydrophobicity; this results in increased sensitivity of the molecular chain conformation to temperature variations. Composite gels that were prepared using highly substituted HBPEC exhibit stronger interactions between hydrophobic alkyl chains, which lead to a lower LCST than for low-substituted gels. The light transmittance curve of the H_0.98–2.32_/P hydrogel is shown in Figure 2A with respect to changes in temperature. Notably, as the temperature approaches the LCST, the composite hydrogel experiences a sharp decline in transmittance. Figure 2B illustrates how LCST changes for the HBPEC/PNIPAM composite hydrogel with respect to the various DSs of HBPEC. Remarkably, as the DS of HBPEC was increased from 0.98 to 2.32, the LCST of the hydrogel decreased from 33.2 °C to 24.1 °C. According to the formula y = 39.2–6.6x (Figure 2B, where y is the LCST and x is the DS of butoxy), hydrogels with different phase transition temperatures can be designed. Compared to the previous literature reports [36,37,38], HBPEC/PNIPAM hydrogels conveniently modulate LCST behavior by varying the DS of HBPEC, and the precise control of LCST is basically attainable through the derived formula. The H_1.98_/P sample with an LCST close to the optimal temperature of the human body was selected to further study the energy-saving and cooling performances of the smart window.

### 2.3. Optical Properties of the HBPEC/PNIPAM Smart Window

**Figure 3:**
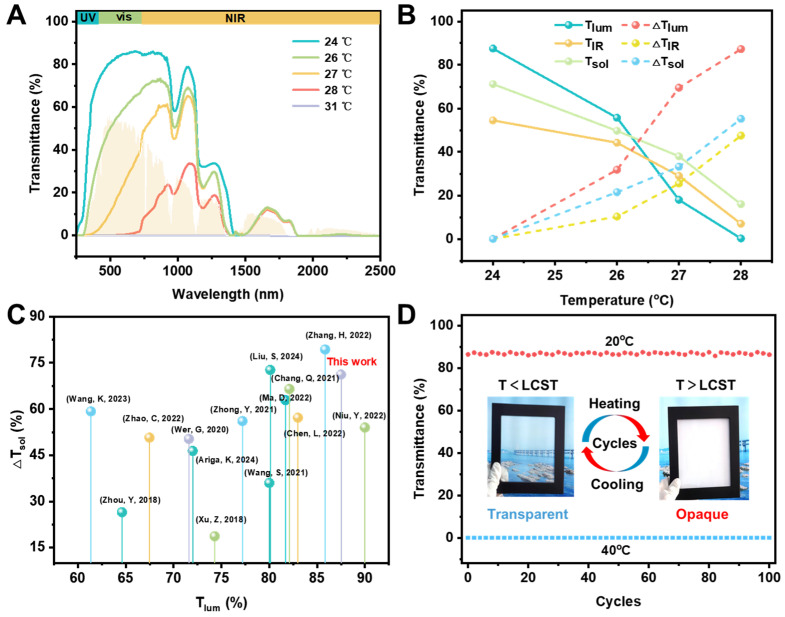
(**A**) UV−vis−NIR spectra of the H_1.98_/P hydrogel smart window at different temperatures. (**B**) Temperature dependence of the H_1.98_/P hydrogel smart window: *T*_lum_, *T*_sol_, *T*_IR_, Δ*T*_lum_, Δ*T*_sol_, and Δ*T*_IR_. (**C**) Comparison with previous work [1,2,3,11,12,13,14,17,22,23,24,25,26,28] (**D**) Cycling stability of H_1.98_/P hydrogels.

Optical transmittance and solar modulation capabilities are also important factors in measuring the performance of smart windows [39,40]. Figure S2 shows the UV–vis–NIR spectrum (200–2500 nm) of the PNIPAM and H_1.98_/P hydrogels at 20 °C. The H_1.98_/P hydrogel exhibits notably higher optical transmittance than the PNIPAM hydrogel. This is because the H_1.98_/P hydrogels have more hydrophilic substances than the pure PNIPAM hydrogels. This increase in hydrophilic substances led to an increase in the number of hydrogen bonds, which in turn increased the transparency of the hydrogels [41,42]. We also investigated the temperature dependence of the H_1.98_/P hydrogel optical properties. Figure 3A shows the UV−vis−NIR transmittance spectrum of the hydrogel over the temperature range of 24–31 °C. At 24 °C, the H_1.98_/P hydrogel smart window has high transparency in the visible and infrared ranges. At higher temperatures, the overall solar transmittance of the H_1.98_/P hydrogel decreases, effectively blocking most solar light from entering the room and preventing a rise in indoor temperature.

Figure 3B and Table S1 show the optical properties of H_1.98_/P at different temperatures. With an increase in temperature (24–31 °C), Δ*T*_lum_, Δ*T*_IR_, and Δ*T*_sol_ gradually decreased. Among them, *T*_lum_ decreased from 87.5% to 0.25%, *T*_IR_ decreased from 54.5% to 7%, and *T*_sol_ decreased from 71.2% to 16%. From a comparison of our work with previous work (Figure 3C), we found that our H_1.98_/P thermochromic smart window exhibited remarkable improvements in both transmittance (Δ*T*_sol_ = 87.5%) and light transmittance (*T*_lum_ = 71.2%).

More importantly, good light modulation stability is also essential for smart windows in practical applications [43]. We tested the transmittance of H_1.98_/P hydrogels after 100 cycles at 20 °C and 40 °C, and the results are depicted in Figure 3D. The transmittance of the hydrogel remained basically the same, which indicates that the H_1.98_/P hydrogel had stable light modulation performance. The inset of Figure 3D is a photograph of the smart window after 100 cycles at 20 °C and 40 °C. At 20 °C, the pattern behind the window can be clearly observed through the H_1.98_/P hydrogel, which indicates that the H_1.98_/P hydrogel has high transparency at room temperature. At 40 °C, the pattern behind the smart window is invisible because the window is almost completely opaque.

**Figure 4:**
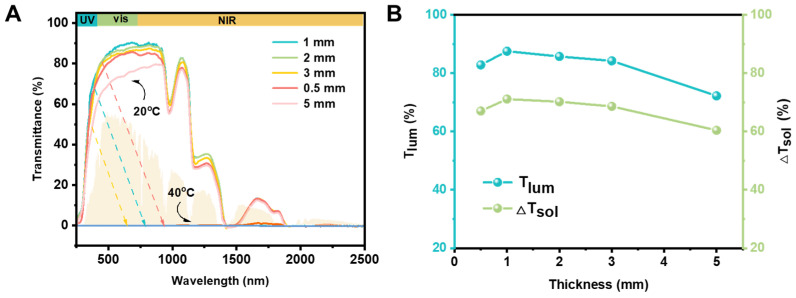
(**A**) Transmittance spectra and (**B**) the *T*_lum_ and Δ*T*_sol_ for 0.5, 1, 2, 3, and 5 mm H_1.98_/P smart windows at 20 °C and 40 °C.

Figure 4A shows the UV−vis−NIR transmittance spectra of the smart windows with different hydrogel thicknesses. High *T*_lum_ and Δ*T*_sol_ of 87.5% and 71.1% are observed for smart windows with a H_1.98_/P of 1 mm (Figure 4B). The Δ*T*_sol_ for 2, 3, and 5 mm H_1.98_/P smart windows are 70.2%, 68.6%, and 60.4%, respectively, and *T*_lum_ declines from 87.5% to 72.2% with the thickness increased to 5 mm. However, as the hydrogel thickness was decreased to 0.5 mm, the interface perturbation and local overheating during in situ free radical polymerization may cause local phase separation [44], which reduced the *T*_lum_ to 82.8% and Δ*T*_sol_ to 67%.

**Figure 5:**
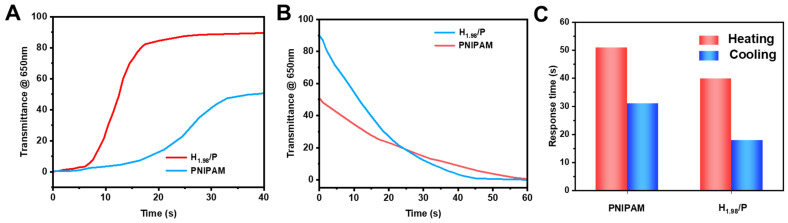
Transmittance@650 nm for pure PNIPAM and H_1.98_/P against heating (**A**) and cooling (**B**) time. (**C**) Response time summary of PNIPAM and H_1.98_/P samples during heating and cooling.

As a smart hydrogel, the response time is one of the key performance indexes. The response times of PNIPAM and H_1.98_/P hydrogels were evaluated by recording their transmittance change in the heating and cooling process (Figure 5A,B). Figure 5C illustrates that, within the temperature range of 20 to 40 °C, the PNIPAM hydrogel takes 51 s to become opaque, while the H_1.98_/P hydrogel achieves opacity in only 40 s. Moreover, PNIPAM needs 31 s to restore transparency, whereas the H_1.98_/P hydrogel achieves this in a mere 18 s. Notably, the HBPEC-modified hydrogel exhibits a faster response speed compared to traditional PNIPAM.

### 2.4. Mechanical Properties of the HBPEC/PNIPAM Hydrogel

**Figure 6:**
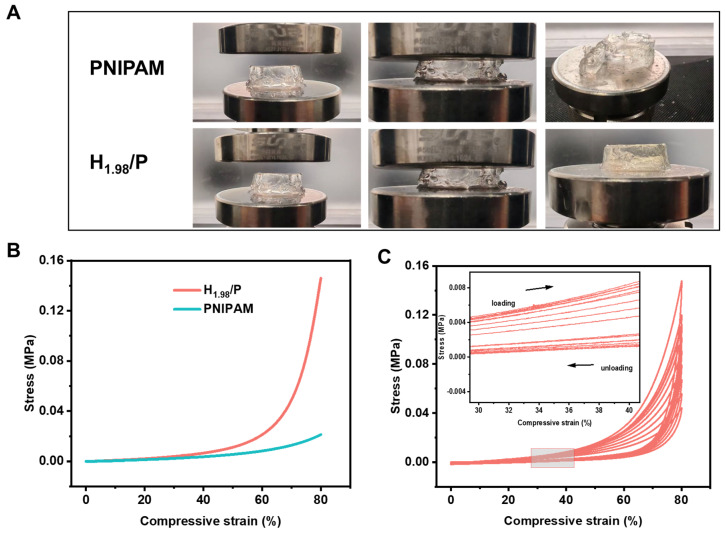
(**A**) Photographs of recovery of mechanical properties of PNIPAM and H_1.98_/P hydrogels. (**B**) Stress–strain curves of PNIPAM and H_1.98_/P hydrogels. (**C**) The 10 cycles of the loading–unloading curves for the H_1.98_/P hydrogel.

Favorable mechanical properties of hydrogels are crucial for providing adhesion and structural support for windows, effectively preventing the hydrogel rupturing and leaking in case a window breaks [45,46]. To assess the applicability of these hydrogels as smart window materials, we subjected PNIPAM and H_1.98_/P hydrogels to 80% strain compression to evaluate their performance. Figure 6A illustrates that the PNIPAM hydrogel underwent significant deformation upon compression and was not able to regain its initial shape. Conversely, H_1.98_/P hydrogels promptly returned to their original columnar form and remained intact after compression; this is favorable mechanical behavior. We also analyzed the stress–strain curves of the PNIPAM and H_1.98_/P hydrogel. Figure 6B shows that H_1.98_/P hydrogels exhibited better compressive stress (increasing from 0.02 MPa to 0.15 MPa) than pure PNIPAM. This enhancement is attributed to the formation of numerous reversible hydrogen bonds between hydrogel molecular chains when HBPEC was incorporated into the hydrogel, resulting in a more compact molecular network structure and improved mechanical strength. Also, the H_1.98_/P hydrogel was tested for 10 load-to-unload compression cycles (Figure 6C). Notably, the hydrogel consistently exhibited a distinct hysteresis loop. Furthermore, the stress–strain curves within the 30–40% compression strain range exhibited excellent overlap over all ten cycles (inset of Figure 6C). It was further demonstrated that the H_1.98_/P hydrogel has stable compression recovery properties and good mechanical properties.

### 2.5. Energy-Saving Performance of the HBPEC/PNIPAM Smart Window

**Figure 7:**
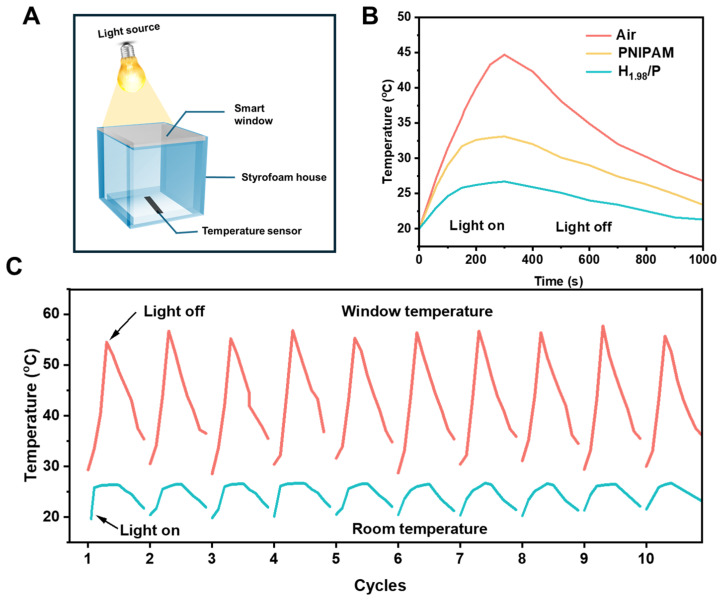
(**A**) Diagram of laboratory simulation tests. (**B**) Changes in indoor temperature of simulations using model houses with three different types of windows that were irradiated using an infrared lamp. (**C**) Time dependence of top window temperature and indoor temperature changes of the H_1.98_/P smart window in response to 10 cycles of infrared lamp irradiation of heating power.

To investigate the energy-saving performance of H_1.98_/P smart windows, we constructed a model house, as shown in Figure 7A. We used identical test conditions, an infrared lamp as a simulated solar irradiation source, and a temperature probe to record internal temperature changes in the model house. The model house was tested using H_1.98_/P, PNIPAM, and ordinary glass during infrared light exposure. The actual device diagram is shown in Figure S3. As shown in Figure 7B, after 300 s of irradiation, the indoor temperature of the model house with ordinary glass and PNIPAM windows rose sharply (44.7 °C and 33.1 °C, respectively). In contrast, light was blocked in the model house with the H_1.98_/P window because of the phase transition of the H_1.98_/P window at 26.3 °C. The indoor temperature of the model house with the smart window composed of the H_1.98_/P hydrogel was only 26.7 °C. The indoor temperature was 18 °C and 6.4 °C lower than that of the model house with ordinary glass and PNIPAM windows, respectively. Clearly, the use of H_1.98_/P hydrogel smart windows resulted in a slower temperature increase in the model house compared to the models with PNIPAM windows and ordinary glass windows.

The long-term performance and stability of smart windows are crucial for practical applications, especially considering their exposure to various hot and cold conditions [47,48,49]. To verify the cyclic stability of the thermotropic phase changes of H_1.98_/P hydrogels, we conducted several infrared lamp heating and cooling tests (Figure 7C). During each cycle, when the surface temperature of the H_1.98_/P smart window reached 26.3 °C, the hydrogel underwent a phase transition, becoming opaque and blocking infrared light. Simultaneously, the room temperature decreased and remained below 26.7 °C. Following 10 cycles, the room temperature stabilized around 26.5 °C under infrared light exposure. This demonstrates the H_1.98_/P smart window’s consistent and reliable light-blocking capability and room temperature regulation.

## 3. Conclusions

In this work, we developed a HBPEC/PNIPAM hydrogel material that was synthesized using low-temperature free radical polymerization, and we confirmed that the LCST of HBPEC/PNIPAM hydrogels can be precisely tuned by varying the DS of HBPEC. Specifically, when the DS of HBPEC was changed from 0.98 to 2.32, the LCST of the hydrogel changed from 33.2 to 24.1 °C. This flexibility enables the customization of materials for catering to diverse temperature requirements. Unlike conventional thermochromic materials, this hydrogel not only demonstrates significant solar modulation (Δ*T*_sol_ = 71.2%) and high transmittance (*T*_lum_ = 87.5%) but is also achieved via a straightforward preparation process and has excellent mechanical properties and long-lasting durability. It is worth noting that after 100 heating/cooling cycles over 20~40 °C, the hydrogel maintains consistent transparency and solar modulation performance, exhibiting stable optical behavior. Furthermore, the HBPEC/PNIPAM smart window exhibits exceptional and consistent indoor temperature regulation capabilities. Therefore, we believe that the novel temperature-responsive hydrogel is a promising candidate for fabricating smart windows that have excellent properties for buildings. Moreover, it holds promising applications in temperature sensors, body temperature monitoring, and thermal management.

## 4. Materials and Methods

### 4.1. Materials

N-Isopropylacrylamide (NIPAM, 98%) monomer was purchased from Macklin, Shanghai, China. N,N′ methylenebis (acrylamide) (MBA, 99%) crosslinkers, potassium persulfate (KPS, ≥98%), and sodium bisulfite (NaHSO_3_) redox initiators were purchased from Aladdin, Shanghai, China. Hydroxyethyl cellulose (HEC) was obtained from Sigma-Aldrich (Burlington, VT, USA, MSOH = 2.5, MW = 3.64 × 10^5^ g/mol, PDI = 2.15). Butyl glycidyl ether (BGE > 99%) was obtained from Tokyo Chemical Industry Co., Ltd. (Tokyo, Japan). None of the chemicals were further purified. Deionized water was used throughout the experiments.

### 4.2. Synthesis of 2-Hydroxy-3-butoxypropyl Hydroxyethyl Celluloses (HBPEC)

In a 100 mL three-necked flask, 2.0 g of HEC (7.3 mmol of anhydroglucose units AGU), 12 mL of deionized water, and 0.8 g of NaOH aqueous solution (40 wt%) were added. The mixture was placed in a 70 °C water bath and stirred for 60 min. A predetermined amount of BGE was added dropwise to the flask. The reaction was continued for 5 h. After this, the reaction system was removed from the water bath and cooled to room temperature. The mixed solution was neutralized with 1 mol/L hydrochloric acid to a pH of 7, and then the mixed solution was poured into a dialysis bag. After dialysis in 8000–14,000 for 72 h, most of the water in the post-dialysis mixed solution was removed using a rotary evaporator and then freeze-dried to residue. (The calculation method for the DS of HBPEC can be found in our previous work and in the Supplementary Materials [50].)

### 4.3. Fabrication of the HBPEC/PNIPAM Hydrogel

First, 0.9 g of NIPAM and 0.2 g of HBPEC were added to 7 g of deionized water and stirred at 70 °C for 60 min to obtain a uniformly dispersed solution, which was then stirred at room temperature to obtain a uniform transparent solution. Next, 5.44 mg of MBA, 5 mg of KPS, and 3 mg of NaHSO_3_ were added to the solution, which was stirred until the contents were completely dissolved. After the last added component (NaHSO_3_) was dissolved, the solution was immediately injected into a pre-cooled laminated glass mold at 0 °C and immersed in an ice water bath until the reaction was complete, thus obtaining the HBPEC/PNIPAM hydrogel.

### 4.4. Fabrication of the Model House and Smart Window

A model room with a size of 18 × 17 × 16.5 cm^3^ was made using foam board. To prepare the HBPEC/PNIPAM smart window, the composite hydrogel precursor solution was injected into a glass gap using a needle tube, and this was sealed well; then, the clamped glass was immersed in an ice water bath until the reaction was complete. Finally, the smart window was assembled in the foam model house. For comparison, air sandwich windows and PNIPAM sandwich windows were also fabricated using a similar method.

### 4.5. Mechanical Characterization

Stress–strain curves were measured using an electrical universal material testing machine (Suns-UTM-4103, Shenzhen, China) with a compression rate of 5 mm/min. A load cell with a max range of 10 kN was attached to the machine. The hydrogels were formed as cylinders with a height of 8 mm and a diameter of 22 mm.

### 4.6. Optical Characterization

The morphologies of the PNIPAM and HBPEC/PNIPAM hydrogels were observed using scanning electron microscopy (SEM, JSM-6010 PLUS/LA, Tokyo, Japan). The infrared spectra of pure PNIPAM, HBPEC, and HBPEC/PNIPAM hydrogels were recorded using a Fourier transform infrared spectrometer (Shimadsu-IR Tracer 100, Kyoto, Japan). The transmittance of the composite hydrogel was measured on an ultraviolet–visible spectrophotometer (V-750, JASCO, Co., Tokyo, Japan) at a wavelength of 550 nm. The temperature at a transmittance of 50% was recorded as the LCST of the composite hydrogel.

A UV–vis–NIR spectrometer (Shimadzu-UV 3600 Plus, Kyoto, Japan) was used to monitor the spectrum under 200–2500 nm incident irradiation. The integral luminous transmittance *T*_lum_ (380–780 nm), IR transmittance *T*_NIR_ (780–2500 nm), solar transmittance *T*_sol_ (300–2500 nm), and the corresponding transmittance modulation can be calculated using Formulas (1) and (2), respectively:

$$T_{lum/IR/sol}=\frac{\int \varphi_{lum/IR/sol}(\lambda )T(\lambda )d\lambda }{\int \varphi_{lum/IR/sol}(\lambda )d\lambda }$$

In the formula, *T*(λ) represents the transmittance recorded at a selected wavelength; *φ*_lum_ is the standard luminous efficiency function of the human eye’s photon vision (Figure S1A); and *φ*_IR_/_sol_ (λ) is the infrared/solar irradiance spectrum of 1.5 air masses (Figure S1B).

$$∆T_{lum/IR/sol}=T_{lum/IR/sol}\left(transparent\;state\right)-T_{lum/IR/sol}(opaque\;state)$$

*T*_lum/IR/sol_ (transparent state) is the integral luminous transmittance/IR transmittance/solar integral transmittance in the transparent state and *T*_lum/IR/sol_ (opaque state) is the integral luminous transmittance/IR transmittance/solar integral transmittance in the opaque state.

### 4.7. Indoor Temperature Regulation Simulation Tests

A simulated house with the HBPEC/PNIPAM smart window and an infrared lamp (Philips, R125, 250W, Amsterdam, The Netherlands) were used to carry out laboratory simulation tests. A temperature probe was used to measure the indoor temperature.

## Figures and Tables

**Table 1:** Components of hydrogels.

Sample	DS	HBPEC (g)	NIPAM (g)	MBA (mg)	KPS (mg)	NaHSO_3_ (mg)	H_2_O (g)
PNIPAM	-	0.2	0.9	5.44	5	3	7
H_0.98_/P	0.98	0.2	0.9	5.44	5	3	7
H_1.32_/P	1.32	0.2	0.9	5.44	5	3	7
H_1.57_/P	1.57	0.2	0.9	5.44	5	3	7
H_1.98_/P	1.98	0.2	0.9	5.44	5	3	7
H_2.32_/P	2.32	0.2	0.9	5.44	5	3	7

## Data Availability

All relevant data are included in the manuscript.

## Supplementary Materials

The following supporting information can be downloaded at: https://www.mdpi.com/article/10.3390/gels10080494/s1, Figure S1: (A) The standard luminous efficiency function graph for the photopic vision of human eyes. (B) Graph of solar irradiance spectrum for air mass 1.5; Figure S2: UV−vis−NIR transmittance spectra of PNIPAM, and HBPEC/PNIPAM hydrogels at 20 °C (the inset is the solar irradiance spectrum, filled area); Figure S3: Indoor simulation experiment device photo; Figure S4: ^1^H-NMR of HBPEC-2 recorded in DMSO-d6; Figure S5: ^13^C-NMR of HBPEC-2 recorded in DMSO-d6; Table S1: Comparison of optical properties of H_1.98_/P hydrogels at different temperatures; Table S2: HBPEC with different DS.
