# Supplementary material for: Energy-Efficient Smart Window Based on a Thermochromic Hydrogel with Adjustable Critical Response Temperature and High Solar Modulation Ability
Source: Gels. 2024 Jul 25;10(8):494. doi: 10.3390/gels10080494 (PMC11353268; doi:10.3390/gels10080494)
Supplement: Supplementary file 1 [file gels-10-00494-s001.zip › gels-3108088-supplementary.pdf]

Supplementary materials

1. Luminescence function of human vision and solar irradiance spectrum

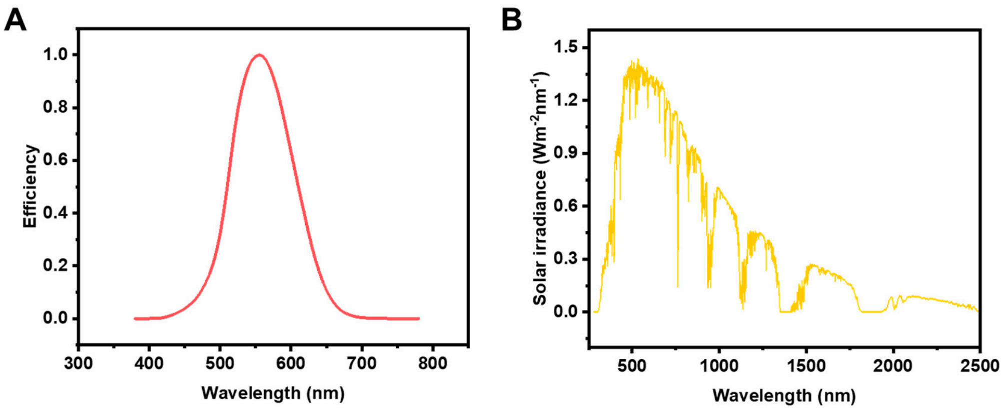

Figure S1. (A) The standard luminous efficiency function graph for the photopic vision of human eyes. (B) Graph of solar irradiance spectrum for air mass 1.5.

2. UV−vis−NIR transmittance spectra

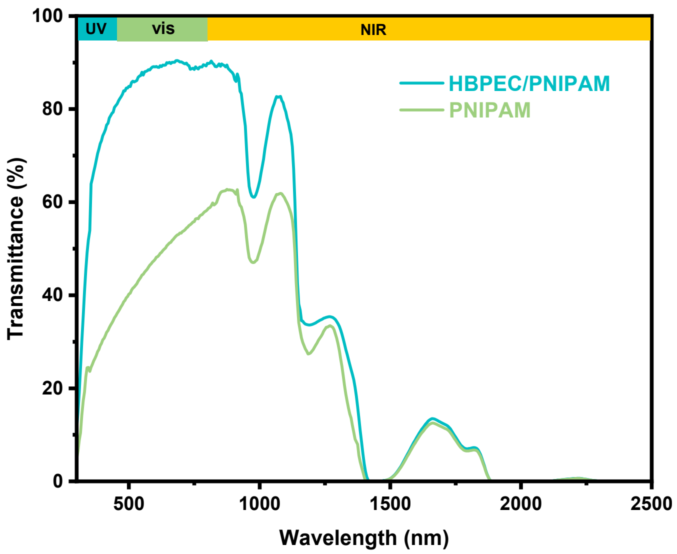

Figure S2. UV–vis–NIR transmittance spectra of PNIPAM, and HBPEC/PNIPAM hydrogels at 20 °C (the inset is the solar irradiance spectrum, filled area).

3. Photograph of the measurement setup

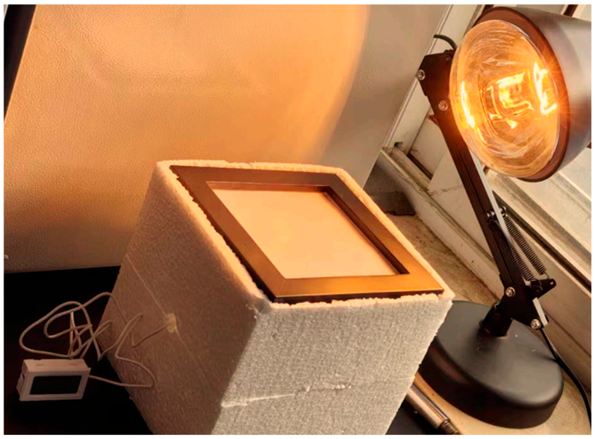

Figure S3. Indoor simulation experiment device photo.

4. The DS of HBPEC product was calculated according to the following formula

$$DS = \frac{\left(\frac{I_{CH3}}{3}\right)}{\left(I_{H1}\right)} \quad (1)$$

In the formula
:

I_{CH3} —— End methyl integral area

I_{H1} —— The integral area of 1-H in AGU

The structure of HBPEC was characterized by ^{1}H NMR and the DS was calculated. Taking HBPEC-2 as a representative (Table S2), ^{1}H NMR and ^{13}C NMR of HBPEC-2 are shown in Figure S4 and Figure S5, respectively. Figure S4 shows the chemical shifts (δ) of 0.86, 1.30, and 1.46 ppm were the proton peaks of the terminal methyl (H15) and methylene (H14 and H13) in the butoxy group. Due to the substitution reaction on the hydroxyl group at the 2-O position, H1 absorption peak of AGU at δ 4.55~4.70 ppm appears as double peaks (H1 and H1`). The broad signal peaks between δ 2.70 and δ 4.00 ppm are the proton peaks of the anhydro glucose unit (AGU) and the O (CH_2CH_2O) x-CH_2-CHOH-CH_2-O-CH_2 group. The MS of HBPEC was determined by ^{1}H NMR. The DS of the HBPEC-2 was calculated by Formula 1

Figure S5 shows peaks at δ 13.64, 18.81, and 31.06 ppm that are related to the terminal

methyl (C15) and methylene (C14 and C13) of the butyl group, respectively. There is a double peak related to C1 at approx. δ 102 ppm. This stems from the cleavage of the hydroxyl group at the 2-O position promoting a substitution reaction, which also promotes the etherification reaction between the reactant HEC and BGE.

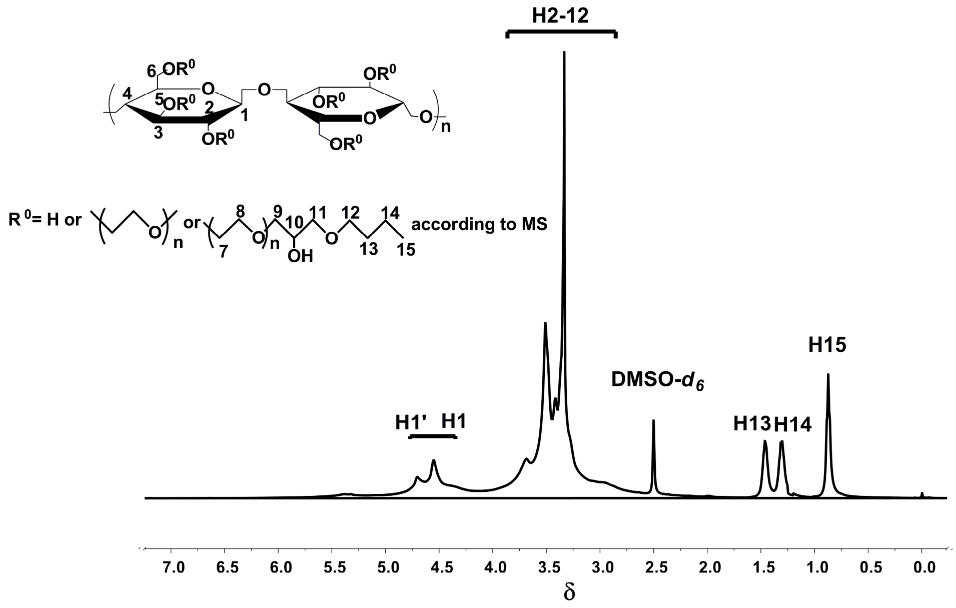

Figure S4 ^{1}H-NMR of HBPEC-2 recorded in DMSO-d6.

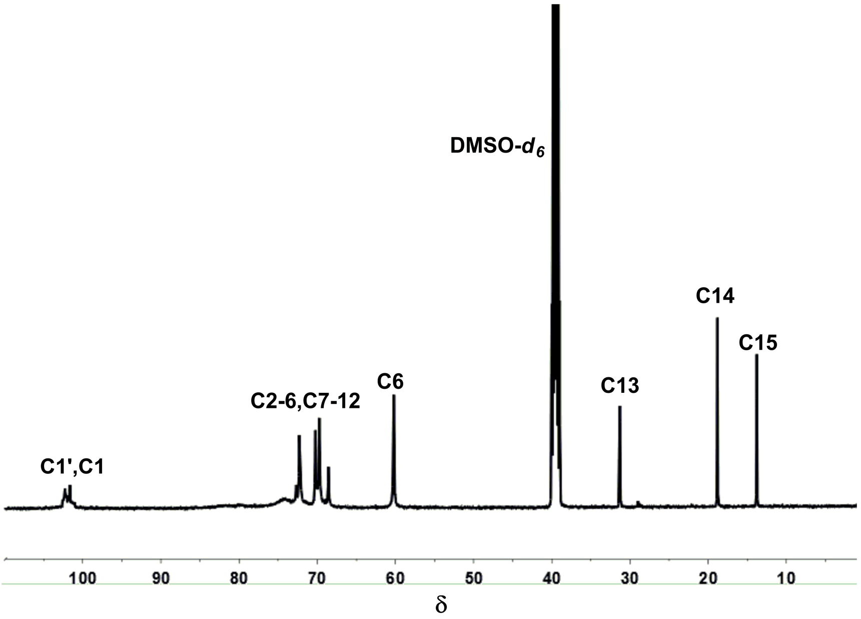

Figure S5 ^{13}C-NMR of HBPEC-2 recorded in DMSO-d6.

Table S1. Comparison of optical properties of $H_{1.98}$/P hydrogels at different temperatures.

Temperature (℃)	T_{lum}	T_{IR}	T_{sol}	$\triangle T_{lum}$	$\triangle T_{TR}$	$\triangle T_{sol}$
24	87.5	54.5	71.2	0.029	0.021	0.026
26	55.7	44.2	49.7	31.8	10.3	21.5
27	18	29	38	69.5	25.5	33.2
28	0.25	7	16	87.25	47.5	55.2

Table S2. HBPEC with different DS

	n(BGE):n(AGU)	DS
HEC	-	-
HBPEC-1	2.0	0.98
HBPEC-2	2.5	1.32
HBPEC-3	3.0	1.57
HBPEC-4	3.5	1.98
HBPEC-5	4.0	2.32
